# Anti-Tumor Effects and Toxicity Reduction Mechanisms of *Prunella vulgaris*: A Comprehensive Review

**DOI:** 10.3390/molecules29081843

**Published:** 2024-04-18

**Authors:** Na Ning, Yi Nan, Guoqing Chen, Shicong Huang, Doudou Lu, Yating Yang, Fandi Meng, Ling Yuan

**Affiliations:** 1 College of Pharmacy, Ningxia Medical University, Yinchuan 750004, China; 20220720319@nxmu.edu.cn (N.N.); 20220720321@nxmu.edu.cn (G.C.); 20220710294@nxmu.edu.cn (S.H.); 2 Key Laboratory of Ningxia Ethnomedicine Modernization, Ministry of Education, Ningxia Medical University, Yinchuan 750004, China; 20080011@nxmu.edu.cn; 3 College of Traditional Chinese Medicine, Ningxia Medical University, Yinchuan 750004, China; 20220130063@nxmu.edu.cn (D.L.); 20210410204@nxmu.edu.cn (Y.Y.); 20220410212@nxmu.edu.cn (F.M.)

**Keywords:** *Prunella vulgaris*, active ingredient, digestive system tumor, mechanism of action

## Abstract

Purpose: To investigate and systematically describe the mechanism of action of *Prunella vulgaris* (*P. vulgaris*) against digestive system tumors and related toxicity reduction. Methods: This study briefly describes the history of medicinal food and the pharmacological effects of *P. vulgaris*, focusing on the review of the anti-digestive tumor effects of the active ingredients of *P. vulgaris* and the mechanism of its toxicity reduction. Results: The active ingredients of *P. vulgaris* may exert anti-tumor effects by inducing the apoptosis of cancer cells, inhibiting angiogenesis, inhibiting the migration and invasion of tumor cells, and inhibiting autophagy. In addition, *P. vulgaris* active ingredients inhibit the release of inflammatory factors and macrophages and increase the level of indicators of oxidative stress through the modulation of target genes in the pathway to achieve the effect of toxicity reduction. Conclusion: The active ingredients in the medicine food homology plant *P. vulgaris* not only treat digestive system tumors through different mechanisms but also reduce the toxic effects. *P. vulgaris* is worthy of being explored more deeply.

## 1. Introduction

Digestive system carcinoma, including esophageal carcinoma, colorectal carcinoma, gastric carcinoma, pancreatic carcinoma, gallbladder carcinoma, liver carcinoma and so on, have a high incidence and mortality [1]. While significant advancements in medical technology have made great progress in cancer treatment, enhancing cancer survival rates and prognostic efficacy remains a formidable challenge [2]. Conventional treatment methods such as surgery, radiotherapy and targeted therapy all cause greater toxic side effects. Chinese medicine has a systematic theory and practical history in cancer and has better therapeutic effects while also reducing the toxic side effects caused by radiotherapy, making it a promising clinical treatment method for tumors [3].

Gastric carcinoma is the third deadliest type of cancer [4]. Its pathogenesis is complex and closely related to age, high salt intake and a lack of fruit and vegetables. *Helicobacter pylori* infection is the main cause of gastric cancer, which has declined with improved food preservation techniques and treatment of *Helicobacter pylori*, but poor prognosis has continued to increase the rate of gastric cancer [5]. Liver carcinoma typically arises from underlying diseases, mainly hepatitis B virus, fatty liver, poor diet and fungal toxins [6], and it is the only one of the grievous carcinoma whose incidence is increasing every year [7]. Its prognosis is poor, with only 5–15% of early-stage patients being suitable for surgical resection, usually without cirrhosis due to the reduced regenerative capacity of the liver, and the efficacy of treatment with kinase inhibitors in patients with advanced disease is extremely low [8]. Colorectal carcinoma is a common malignancy of adenocarcinoma origin, whose incidence increases with age; in about 80% of cases, patients are diagnosed with localized tumors, for which the causative factors are mainly age and family history. Surgery is the main treatment for localized disease, while post-operative chemotherapy serves to reduce recurrence, especially if the lesion has spread to the lymph nodes. Notably, a combination of chemo-radiotherapy serves as the main therapeutic approach for rectal carcinoma other than surgery and is often administered preoperatively [9]. Gallbladder carcinoma is usually diagnosed late in the advanced stages, characterized by substantial tumor size leading to obstruction of and infiltration into adjacent tissues and organs. In most cases, diagnosis occurs incidentally during surgical intervention for gallstones, highlighting the lack of routine screening or pro-phase diagnostic protocols for early detection unlike several other cancers [10]. Despite the qualitative progress in the index of existence for other carcinomas, the index of existence for pancreatic carcinoma has been almost unchanged since the 1960s. The disease is often diagnosed at a late stage, with many treatment methods exhibiting limited efficacy, resulting in a discouraging prognosis, with influencing factors that are strongly correlated with genetics, obesity, alcohol and diabetes, among others [11]. Esophageal carcinoma has an increased lethality ratio. The main factors include smoking, alcohol consumption and pancreatic dysphagia, with simple dysphagia or unexpected weight loss being the most common first symptoms [12].

Indigenous cultures around the world have long used traditional herbal medicine to treat a myriad of ailments, with plants gaining recognition for their therapeutic properties, and this proliferation has produced highly effective medicines for widespread clinical use, including many plant natural products and derived analogues, among others [13]. The discovery and development of natural complexes, valid constituents and formulations in herbal medicines with the option for a cure that can prioritize the destruction of tumor cells and suppress carcinoma proliferation, as well as toxicity reduction, is a critical field of carcinoma remedy. Cancer is a multifactorial and multistep disease, so the therapeutic effect of combined herbal formulas with multiple targets and signaling pathways on tumors may be superior to that of drugs acting on a single molecular target [14]. Studies have indicated that edible plants can decrease the probability of illness, especially cancer. China has explored a variety of traditional Chinese medicines through long-term practice in history and summarized their application to different diseases [15]. Some traditional Chinese medicines can be used for both medicine and food, that is, medicine and food are homologous [16]. Its therapeutic effect is due to the effective chemical constituents of plants [17]. Modern pharmacological studies have shown that the broad pharmacological effects of *P. vulgaris* mainly contain anti-inflammatory, antioxidation, anti-hypertension, anti-hyperlipidemia, immune regulation, anti-virus and anti-carcinoma functions [18]. It also has a crucial impact on the fight against digestive system tumors. It can be used to prevent and treat gastric carcinoma [19], liver carcinoma [20], colorectal carcinoma [21], pancreatic carcinoma [22], gallbladder carcinoma, esophageal carcinoma and other malignant tumors [23].

*P. vulgaris*, commonly known as “Xiakucao” in China, is a perennial herb. *P. vulgaris* belongs to the Lamiaceae family [24]. *P. vulgaris* is a highly valuable medicinal plant that is widely distributed and adapts to different geographic environments, such as China, Korea, Japan and Europe [25]. It has been shown to be used to treat sore throats and promote wound healing [26]. *P. vulgaris* has been widely recognized for its various active ingredients and remarkable pharmacological effects, showing great potential for clinical applications [27]. This paper provides a brief overview of the history and pharmacological effects of *P. vulgaris*. It also systematically reviews the active ingredients in relation to digestive system tumors and discusses mechanisms for reducing toxicity. As a plant with both medicinal and culinary uses, *P. vulgaris* shows promise for further development.

## 2. Methodology

**Figure 1:**
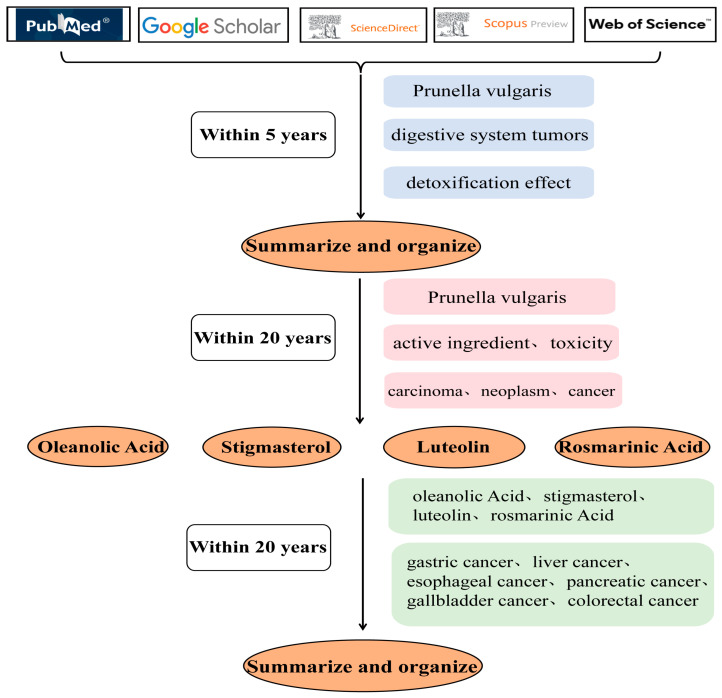
Literature retrieval process.

Firstly, the keywords “*P. vulgaris*”, “digestive system tumors” and “detoxification effect” were used in PubMed, Google Scholar, ScienceDirect, Scopus and Web of Science databases, and the search time was set to within 5 years. It was found that there was no review on the treatment of digestive system tumors with *P. vulgaris* in the past five years, and only a few articles were on the direction of tumors. The whole article only reviewed breast neoplasm, thyroid neoplasm, liver neoplasm, lung neoplasm or colon neoplasm. Secondly, the keywords “*P. vulgaris*”, “active ingredient”, “carcinoma”, “neoplasm”, “cancer” and “toxicity” were used to search the literature within 20 years. Finally, “*P. vulgaris*”, “oleanolic Acid”, “stigmasterol”, “luteolin”, “rosmarinic Acid”, “gastric cancer”, “liver cancer”, “esophageal cancer”, “pancreatic cancer”, “gallbladder cancer” and “colorectal cancer” were used as keywords, and we set the search time to be within 20 years. We systematically reviewed the intervention of active components of *P. vulgaris* in digestive system tumors, including not only different types of cancers in the digestive system but also the corresponding targets, signal axes and tumor phenotypes of each cancer type. The literature retrieval process is shown in Figure 1.

## 3. History of Medicine and Food

As the earliest record of medicinal use, *P. vulgaris* was found in the “Shen Nong’s Herbal Classic” of the Han Dynasty. *P. vulgaris* is bitter, pungent and cold in nature. It has many effects [28], such as “main heat scrofula, rat fistula, head trauma, broken disease, scattered galls, knot gas, swollen feet, wet arthralgia, clearing liver and gallbladder”. *P. vulgaris* was first recorded to be edible in the Song Dynasty “Bencao Yanyi”: “*P. vulgaris*... When the young leaves are used as vegetables, they must be washed to remove bitter water”. It is worth mentioning that Yao Kecheng compiled the “Food Materia Medica” in the Ming Dynasty. This collection is a monograph on diet conditioning and food treatment before the Ming Dynasty in China. *P. vulgaris* is also included: “*P. vulgaris*, bitter, cold, non-toxic... The tender seedlings are reduced, the bitterness is soaked, and the oil and salt are mixed to make the crispy and beautiful”. Li Shizhen made a detailed record of the medicinal value of *P. vulgaris* in the “Compendium of Materia Medica”. At the same time, he also explained the eating suggestion of *P. vulgaris*: “the young seedlings are reduced, the bitter taste is soaked, and the oil and salt are edible”. It can be seen that *P. vulgaris* has been widely consumed as food and vegetables as early as thousands of years ago [29]. *P. vulgaris* has the effects of detumescence and relieving heat. As a drug, it is also gradually integrated into the upsurge in traditional Chinese medicine health preservation. It has been developed into *P. vulgaris* tea, *P. vulgaris* porridge, cool *P. vulgaris* and so on [30]. *P. vulgaris* has a variety of dosage forms for clinical application, such as tablets, ointments, capsules, granules, etc. However, due to its different extraction methods and preparation processes, its clinical efficacy is also different. It has been proven to have good application prospects and research value in breast, thyroid, tumor and other diseases [31].

## 4. Active Ingredient Related Target Pathway Network Diagram

**Figure 2:**
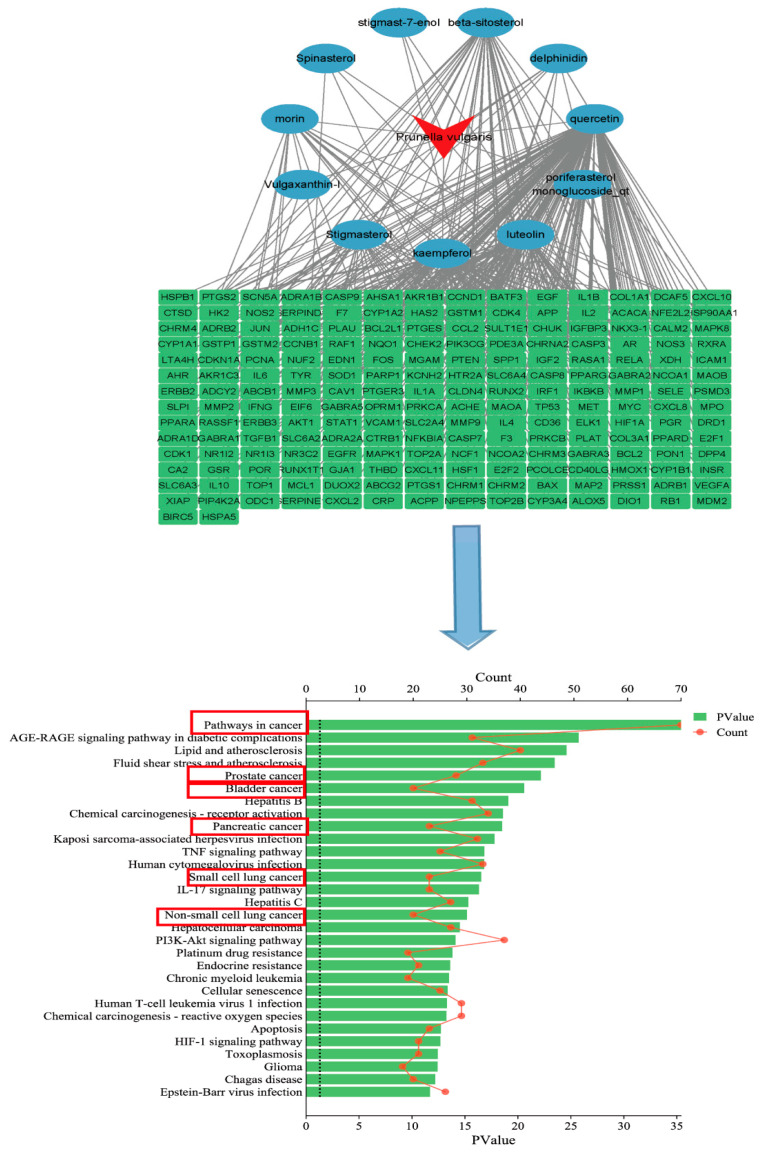
Target acquisition and related pathway screening of *P. vulgaris*.

The effective ingredients of *P. vulgaris* were searched using TCMSP (https://old.tcmsp-e.com/tcmsp.php), accessed on 15 May 2023. With oral bioavailability (OB) ≥ 30% and drug-like properties (DL) ≥ 0.18 set as the select range, the effective ingredients and their relevant target proteins with high activity were screened, the objective protein designations were entered into UniProt (https://www.uniprot.org/), accessed on 15 May 2023 to gain the corresponding target gene designations, and all the obtained gene names were merged and deduplicated. The predicted targets of *P. vulgaris* were embed into David (https://david.ncifcrf.gov/tools.jsp), accessed on 15 May 2023 for Kyoto Encyclopedia of Genes and Genomes (KEGG) channel enrichment analysis. The top 30 signaling channels were screened according to the *p*-value. According to the screening conditions of the *p*-value and Count value, the enrichment analysis results were visualized in the form of a histogram by using the micro-bioinformatics online platform. The results showed that the number of genes enriched to pathways in cancer was the largest, and the *p*-value was the most significant. The screened active components of *P. vulgaris* and their corresponding target genes were used to establish an effective component–drug–target network analysis diagram through Cytoscape 3.9.1 software. Target acquisition and related pathway screening of *P. vulgaris* is shown in Figure 2.

## 5. Active Ingredient

Various bioactive components were identified in the extract of *P. vulgaris* [32]. The main active components were triterpenes [33], saponins [34], sterols, flavonoids [35], phenylpropanoids [18], polysaccharides [36] and volatile oils.

### 5.1. Triterpenes and Their Saponins

A total of 28 triterpenoids were isolated from *P. vulgaris*, including 20 triterpenoid sapogenins and 8 saponins [37]. The highest content was oleanolic acid, which was significantly correlated with the pharmacological effects of *P. vulgaris*. In nature, the compound exists in the structure of a free acid or a triterpenoid saponin glycoside ligand precursor, where it can be combined with sugar chains [38]. The existence of α-l-rhamnose residues at the C-3\28 ends of oleanolic acid double-stranded glycosides is important for enhancing cytotoxicity, and the introduction of further sugar at C3-OH and C-28 carboxylic acid is an advantageous decoration to improve defense against tumor function [39].

### 5.2. Sterols

The sterol compounds in *P. vulgaris* mainly include β-sitosterol, stigmasterol and ∆7-stigmasterol. The structure of plant sterols is similar to that of cholesterol. A double bond consisting of three cyclohexane rings and one cyclopentane ring was found at the C5-6 position. It has been indicated that phytosterols can induce apoptosis in some carcinoma cell lines and exert anticancer activity [40].

### 5.3. Flavonoids

*P. vulgaris* also includes luteolin, iso-orientin and luteoloside. Luteolin is a tetrahydroxy flavonoid compound composed of a carbon structure and two benzene rings connected by heterocycles. It mainly exists in the form of glycoside ligands or glycosides. Luteolin has been found to have the external and internal mechanisms of apoptotic cell mortality in carcinoma. The inhibition of neoplasm cell metastasis and angiogenesis is a potential influence of luteolin and has the possibility of reversing the drug resistance of neoplasm cells [41].

### 5.4. Phenylpropanoids

**Figure 3:**
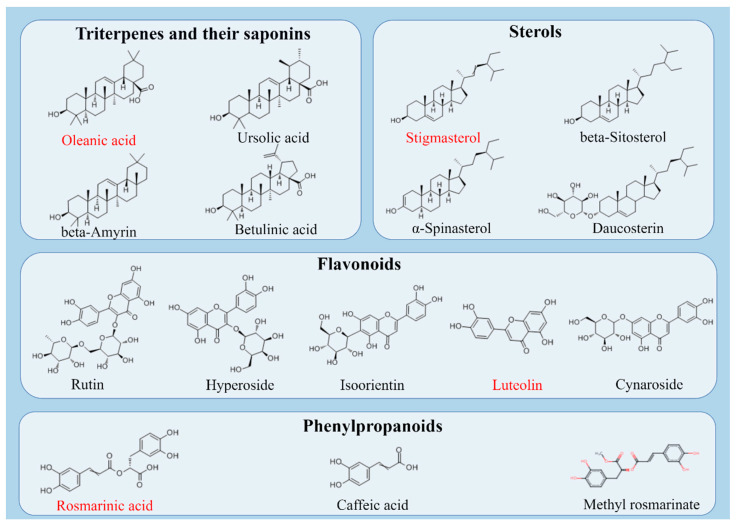
Structural formula of active components in *P. vulgaris*: the compounds highlighted in red are the four active ingredients that were the focus of the review in this study. (Created via Figdraw).

Phenylpropanoids in *P. vulgaris* are cis- and trans-caffeic acids and rosmarinic acid [42]. Among them, rosmarinic acid has been studied more. In industry, rosmarinic acid can be manufactured via esterification concerning 3,4-dihydroxyphenyllactic acid and caffeic acid [43], which has been proven to have a strong anti-tumor role [44]. The structural formula of the active components of *P. vulgaris* is shown in Figure 3.

## 6. Pharmacological Actions

*P. vulgaris* is a plant source of a variety of medicinal active ingredients [45], and its wide range of uses can treat various diseases, including cancer. It has anti-inflammatory effect. The wound-healing effect of the chemical compound of *P. vulgaris* in rats and mice was studied via wound models. The experimentation studies have indicated its remarkable ability to heal a wound and its anti-inflammatory efficacy [46]; antibacterial effect studies have shown that *P. vulgaris* extract has antibacterial activity against Escherichia coli in patients with urinary tract infection [47], as *P. vulgaris* extract is a natural antioxidant that restores the level of the oxidation index and has significant antioxidant and hepatoprotective activities [48]; and studies of anti-hypertensive effects have shown that these effects can significantly reduce systolic blood pressure and blood pressure in rats, with the anti-hypertensive effect having a dose–effect relationship. The acute and chronic hypoglycemic effects of *P. vulgaris* in type I diabetes were evaluated in a mouse model, and studies showed that serum insulin in mice increased and α-amylase and α-glucosidase decreased under the intervention of *P. vulgaris*; therefore, the active ingredients in *P. vulgaris* may be potential drugs to improve type I diabetes and related complications [49]. The effect of anti-hyperlipidemia was evaluated via metabolomics in high-fat rats, and the results showed that *P. vulgaris* significantly reduced the specific gravity of the body, Total Cholesterol, low-density lipoprotein, high-density lipoprotein, malondialdehyde and abdominal fat mass in rats; increased glutathione peroxidase activity; and improved lipid metabolism disorder in rats to interfere with hyperlipidemia [50]. For the immunomodulatory effect, the heteropolysaccharide isolated from the fruit cluster of *P. vulgaris* showed stable immune activity in further research and could be used as an effective immunomodulator in functional food and pharmacology [51]. Regarding antiviral effects, *P. vulgaris* extract can enhance the activity of monoclonal antibodies against Ebola virus and provide new ideas for the development of anti-Ebola virus infection [52]. Regarding anti-tumor effects, *P. vulgaris* inhibits the metastasis of various tumor cells and promotes apoptosis through different pathways [53]. Studies have shown that the *P. vulgaris* polysaccharide complex effectively inhibits the progression of liver carcinoma cells by promoting apoptosis, manifested as cell cycle arrest, chromatin condensation and morphological changes [54]. *P. vulgaris* can regulate various signaling pathways and, thus, improve the microenvironment of liver carcinoma metastasis, so it has the therapeutic potential to suppress the migration and invasion of hepatocellular carcinoma cells [55]. Colon carcinoma cells treated with extracts from the spicate plants of *P. vulgaris* showed a remarkable decrease in cell growth activity [21]. The extracts from *P. vulgaris* inhibited the proliferation of gastric cancer cells by increasing the levels of BAX and decreasing Bcl-2 levels [19] and suppressed the proliferation of gastric carcinoma by downregulating vascular endothelial growth factor (VEGF) expression [56].

## 7. Anti-Tumor Effect of Active Ingredients

**Figure 4:**
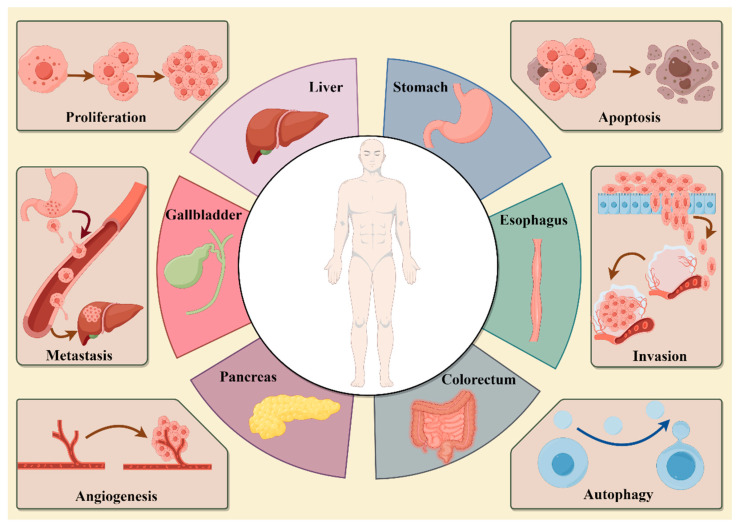
Types and Phenotypes of *P. vulgaris* against digestive system tumors (Created via Figdraw).

The anti-tumor mechanisms of *P. vulgaris* include inhibiting cell proliferation, promoting carcinoma cell apoptosis, promoting autophagy, adjusting cell cyclin, suppressing vascular endothelial growth against angiogenesis and restraining carcinoma cell migration and invasion [23]. Types and Phenotypes of *P. vulgaris* against digestive system tumors is shown in Figure 4.

### 7.1. Oleanolic Acid

As one of the pentacyclic triterpenoids, oleanolic acid has positive anti-tumor activity. The mechanism comprises the following aspects: the suppression of cell proliferation, promotion of apoptosis, facilitation of autophagy, regulation of cell cyclin, suppression of vascular endothelial growth, suppression of angiogenesis, restriction of tumor cell migration and infiltration, etc. [57]. Oleanolic acid has been shown to inhibit a variety of molecular targets that play a crucial function in the progression of carcinoma. In recent years, several synthetic oleanolic acid derivatives have been synthesized, which showed strong anti-tumor properties [58]. The suppression of cell dynamism by OA is caused though the apoptosis of gastric carcinoma cells and the S-stage block of SGC7901, thereby restraining gastric carcinoma cells by suppressing the Protein kinase-B (Akt)/Nuclear factor kappa-B (NF-κB) channel [59]. Oleanolic acid promotes G2-/M-stage stagnation and the apoptosis of hepatocellular neoplasm cells. By diminishing the level of cyclin B1 and regulating the phosphorylation of protein kinase Chk1 and p21, the level of Bcl-2 was significantly reduced, and the BAX, cytoplasmic Cytochrome-C (Cyt c), Caspase-9 and Caspase-3 levels were increased to produce an anti-tumor effect [60]. The combination of oleanolic acid and 5-FU can collaboratively enhance the cell death of pancreatic neoplasm cells and increase the pro-apoptotic effect. Further studies have shown that combination therapy can strengthen mitochondrial depolarization, lysosomal membrane permeability and cathepsin D percolation and reduce the levels of Bcl-2 and survivin [61].

### 7.2. Stigmasterol

Stigmasterol has anti-neoplasmic role by boosting neoplasmic cell apoptosis; restraining growth, metastasis and invasion; and facilitating tumor cell autophagy against carcinoma effects on a variety of malignant tumors. According to mechanism studies, stigmasterol causes neoplasm cell apoptosis by inhibiting the Phosphatidylinositol 3-kinase (PI3K)/AKT pathway and producing mitochondrial reactive oxygen species. Its active role principally depends on its regulation of cyclin, and there are many potential influences on the regulation of cellular activities [62]. Stigmasterol inhibited the growth of gastric cancer cells. Its anti-proliferative effect was due to the induction of mitochondrial-mediated apoptosis, a diminution of BAX and elevation of Bcl-2 levels. It was further confirmed that stigmasterol also suppressed neoplasm cell migration and facilitated G2/M cell cycle stagnation. In addition, stigmasterol can regulate the JAK/STAT channel [63] to exert anticancer activity. Research has shown that stigmasterol inhibits lung neoplasm cell reproduction and promotes apoptosis. By directly targeting stigmasterol in lung cancer by RAR-related orphan receptor C (RORC), RORC overexpression reverses the inhibitory effect of stigmasterol on lung cancer [64].

### 7.3. Luteolin

Luteolin is a naturally occurring polyphenolic compound known to have antioxidant and anti-tumor activities in vitro. It is regarded as an anti-neoplasmic active ingredient to cure various forms of malignant tumors, such as lung carcinoma, gastric carcinoma, colon carcinoma and pancreatic carcinoma. It also prevents the progression of cancer by inhibiting the proliferation of carcinoma cells, protecting against neoplastic irritation, blocking the cell cycle and boosting apoptosis via diverse channels. In addition, it increases the levels of intracellular reactive oxygen species (ROS), PKR-like endoplasmic reticulum kinase (PERK), C/Ebp-Homologous Protein (CHOP), recombinant activating transcription factor 4 (ATF4) and Caspase 12 in glioblastoma cells via the activation of the lethal endoplasmic reticulum stress response and mitochondrial dysfunction and through the activation of endoplasmic reticulum stress-related protein reflection [65]. Luteolin led to the inactivation of cyclin B, Cell-division-cycle kinase 2 (CDC2), Caspase-9 and other proteins in the mouse colon HCT-15 cell line model; upregulated apoptotic protease activating factor-1 (APAF-1), Cyt.C, caspase-9/3 and cyclin-dependent kinase (CDK); arrested the G2/M stage of the cell cycle; and ultimately led to apoptosis and exerted anticancer activity [66].

### 7.4. Rosmarinic Acid

**Figure 5:**
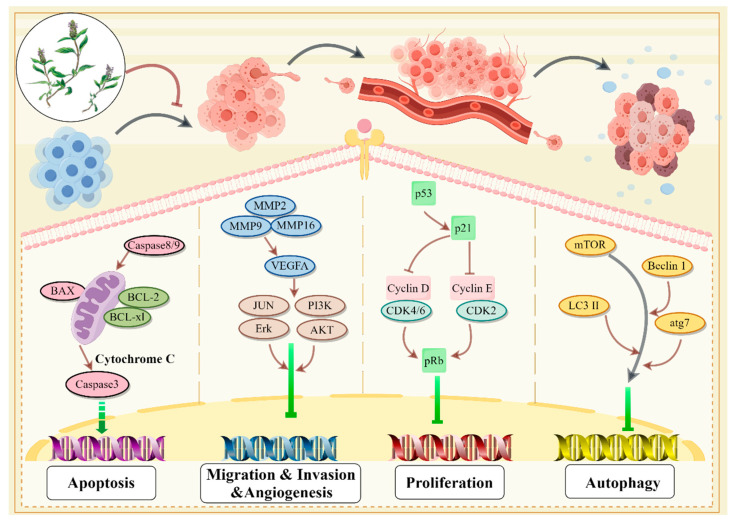
Possible mechanism of *P. vulgaris* against digestive system tumors (Created via Figdraw).

Rosmarinic acid is a common labiatae plant, and its rich nutritional properties are used to improve health. Studies have found that rosmarinic acid can prevent tumorigenesis, inhibit tumor growth and act as an adjuvant therapy to treat sensitization caused by radiotherapy and chemotherapy drugs [67]. In vivo experiments on mouse colon cancer indicated that rosmarinic acid significantly restrained the progression of cancer cells by inhibiting NF-κB and STAT3 [68]. Studies have shown that rosmarinic acid can inhibit NF-κB p65 in the xenotransplantation microenvironment and effectively inhibit the progression of liver carcinoma cells by regulating the secretion of inflammation- and angiogenesis-related factors [69]. The anti-gastric cancer activity of anti-MUC1 and rosmarinic acid is reflected in the inhibition of mRNA expression of enzymes that produce cancer-associated antigens and specific antigens; the promotion of BAX, Bad and Caspase-3 and -9; and inhibition of Bcl-2 expression [70]. Studies have shown that rosmarinic acid blocks the G2/S cycle in pancreatic carcinoma cells by adjusting the communication of cyclin E, BAX and Bcl-2; inhibits the migration and invasion of pancreatic cancer cells through E-cadherin and Matrix metallopeptidase 9 (MMP-9); and inhibits tumor growth [71]. Possible mechanism of *P. vulgaris* against digestive system tumors is shown in Figure 5 and Table 1.

## 8. The Attenuated Effect of Active Ingredients

Other active ingredients in *P. vulgaris* also have protective effects on liver, kidney and lung injury.

**Figure 6:**
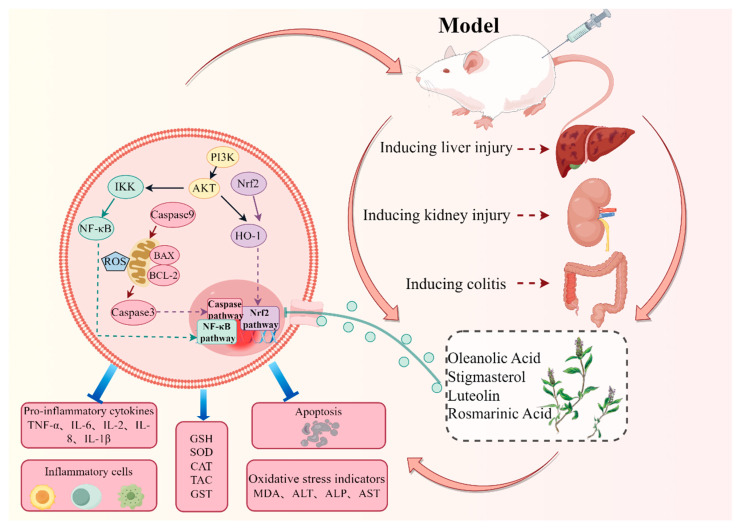
Possible mechanisms of *P. vulgaris* that reduce toxicity (By Figdraw).

Oleanolic acid can reduce liver ischemia-reperfusion injury. The protective mechanism is correlated to the Heme oxygenase-1 (HO-1)/Recombinant Sestrin 2 (Sesn2) channel induced by oleanolic acid, which upregulates Sesn2, PI3K, Akt and HO-1 in the liver and protects the liver [90]. Stigmasterol ameliorates neuroinflammation in mice and inhibits microglial inflammation by relying on AMP-activated protein kinase (AMPK)/NF-κB and AMPK/NOD-like receptor thermal protein domain-associated protein 3 (NLRP3) oligomers. Its function also inhibits neuroinflammation by diminishing proinflammatory element levels and microglial sensitization [91]. Luteolin is a strong anti-inflammatory agent that inhibits NF-κB, Mitogen-activated protein kinase (MAPK), Interleukin 6 (IL-6), Interleukin 8 (IL-8) and TNF-α and reduces ROS content. Luteolin can chelate transition metal ions responsible for ROS production, thereby inhibiting lipoxygenase. It has been proven that the suppression of NF-κB channel by luteolin stimulates the expression of inducible proinflammatory enzymes such as inducible nitric oxide synthase (iNOS), cyclo-oxygenase-2 (COX-2) and IL-1β, IL-6 and TNF-α [92]. Rosmarinic acid can remove active superoxide and peroxynitrite; reduce hepatotoxicity; inhibit the proliferation of hepatic stellate cells; downregulate the activity of transforming growth factor-β1 (TGF-β1), connective tissue growth factor (CTGF) and α-Smooth muscle actin (α-SMA); and improve liver tissue indexes [93]. Possible mechanisms of *P. vulgaris* that reduce toxicity is shown in Figure 6 and Table 2.

## 9. Discussion

*P. vulgaris* is a traditional Chinese medicine that is rich in nutrients and active ingredients as a medicinal and edible plant [105]. *P. vulgaris* not only acts as a cure for diseases but can also be consumed as a nutrient [106]. The active components in *P. vulgaris* plants have extensive biological functions, including anti-inflammatory, antioxidation, anti-hypertension, anti-hyperlipidemia, immunomodulation, anti-virus and anti-tumor. Cancer is a malignant disease that causes human death. The extremely high morbidity and fatality rate consumes a lot of medical resources and is a huge social and economic burden [107]. The active ingredients of *P. vulgaris* especially play an active role in the treatment of digestive system tumors, including stomach, liver, colorectal, gallbladder, pancreatic, esophageal and other malignant tumors. Oleanolic acid, stigmasterol, luteolin and rosmarinic acid in *P. vulgaris* can effectively inhibit tumor progression, and the possible mechanism is to inhibit tumor progression by regulating the expression of target genes, including inducing the apoptosis of cancer cells, inhibiting angiogenesis, inhibiting the migration and invasion of tumor cells, and inhibiting cellular autophagy.

The active ingredients of *P. vulgaris* Cao not only have significant efficacy as anti-tumor agents but also reduce liver and kidney damage and play a protective role. The possible mechanisms of its attenuating action are regulating the expression of target genes in the mitochondrial apoptotic pathway to inhibit apoptosis, inhibiting the release of inflammatory factors and macrophage activation by inhibiting the NF-κB signaling pathway and Nrf2 signaling pathway, and, on the contrary, increasing the levels of oxidative stress indicators such as GSH, SOD, CAT, TAC, GST and so on. Surgery, radiotherapy and chemotherapy are effective ways to treat tumors, but they are often accompanied by large toxic side effects that reduce patient compliance. In this study, we reviewed the anti-tumor effects and toxicity reduction mechanisms of *P. vulgaris*, which exerts its efficacy while minimizing hepatic and renal injuries as much as possible. Its high efficiency and low toxicity may make it a promising clinical treatment modality for tumors.

*P. vulgaris* is widely used in medicine and food as a promising economic medicinal material and functional food. The secondary metabolites of medicinal and edible homologous plants are terpenoids, flavonoids, coumarins, volatile oils, saponins, sterols, etc., which have been confirmed to have a diversified pharmacological activity. They can be combined for multi-target and multi-channel, with good medicinal effects and small side effects [108]. At present, the related research on *P. vulgaris* is not comprehensive and specific enough, and its quality control, plant identification, toxicology and pharmacokinetics are relatively lacking. Therefore, we should further improve the weak part, based on the superiority of the multi-component and multi-target properties of traditional Chinese medicine to clarify the pharmacological effects and mechanisms of traditional Chinese medicine plants and conduct in-depth research on the intervention of traditional Chinese medicine plants in cancer research. To explore its potential nutritional and medicinal value, we have provided a theoretical and scientific basis for subsequent research and supplied a substantial evaluation of the value of *P. vulgaris* in the field of medicine and food.

## Figures and Tables

**Table 1:** The anticancer active constituents and their mechanisms of action of *P. vulgaris* are based on the suggestion that compounds previously identified in *P. vulgaris*.

Component	Tumor	Experimental Model	Mechanism	Action Target	Phenotype	References
Oleanolic Acid	Gastric cancer	Gastric cancer cell lines SGC-7901 and MGC803	NF-κB axis	Blocked G2/M phase, upregulated Bcl-2 and Beclin 1 and downregulated the reflection of BAX and ATG 5, inducing apoptosis and autophagy.	Apoptosis Autophagy	[72]
	Colorectal cancer	Human Colorectal carcinoma cell line HCT-116	MEK/ERK/JNK axis	Inhibited VEGFR2 phosphorylation and tumor angiogenesis.	Angiogenesis	[73]
	Liver cancer	Human Liver cancer cell HepG2	Mitochondrial apoptosis pathway	Blocked the G2/M phase; upregulated Caspase3, BAX; and Cyt. C; and downregulated Bcl-2 expression, inducing tumor cell apoptosis.	Apoptosis	[74]
	Gallbladder cancer	Human Gallbladder cancer cell lines GBC-SD and NOZ	AKT/ERK axis	Blocked G0 phase and downregulated PCNA, ICAM-1 and RhoA to inhibit tumor development.	Proliferation	[75]
	Pancreatic cancer	Human Pancreatic cancer cell line PANC-28	Mitochondrial apoptosis pathway	Blocked G1 phase and G2/M phases, inducing ROS production and Cyt. C release, and activated the lysis of caspases-3/9 and PARP, inducing cell apoptosis.	Apoptosis	[76]
Stigmasterol	Gastric cancer	Gastric cancer cell lines SGC-7901 and MGC803	AKT/mTOR axis	The expressions of BAX, Caspase-3, PARP and LC3-II were upregulated, while the expression of Bcl-2 was downregulated, inducing apoptosis and autophagy.	Apoptosis Autophagy	[77]
	Liver cancer	Human Liver cancer cell HepG2	Mitochondrial apoptosis pathway	Blocked G2/M phase, BAX and p53 were upregulated, Bcl-2 and XIAP were downregulated and aspase-8/9 was activated, inducing apoptosis.	Apoptosis	[40]
	Colorectal cancer	Human Colorectal cancer cell line HCT-116	-	Decline in the expressions of Bcl-2, cIAP-1 and mRNA; elevation in BAX and Mrn;, and promotion of the release of Cyt.C, inducing cell apoptosis.	Apoptosis	[78]
	Gallbladder cancer		Mitochondrial apoptosis pathway	Upregulation of p27 expression, downregulation of Jab1 gene and activation of Caspase-3, inducing cell apoptosis.	Apoptosis	[79]
Luteolin	Gastric cancer	Gastric cancer cell line SGC-7901	cMet/Akt/ERK axis	Reduction in the reflection and phosphorylation of MMP9 and cMet and increase in the reflection of Caspase-3 and PARP-1, inducing apoptosis and reduce invasion.	Apoptosis Invasion	[80]
	Colon cancer	Human Colon cancer SW620 cells	ERK/FOXO3a axis	Decrease BAX, Caspase-3, PARP, and FOXO3a, increase Beclin-1, Atg5, and LC3B-I/II; Induce apoptosis and inhibit autophagy.	Apoptosis Autophagy	[81]
Rosmarinic Acid	Colorectal cancer	Human Colorectal cancer cell line HCT-116	Nrf2/ARE/HO-1 axis	Upregulation of Nrf2 transcription activity, enhancement of HO-1 expression and suppression of proliferation.	Proliferation	[82]
	Liver cancer	Human Liver carcinoma cell HepG2	-	Upregulation of BAX and p53, downregulation of Bcl-2 expression and induction of PARP lysis, inducing apoptosis.	Apoptosis	[83]
	Esophageal cancer	Esophageal carcinoma cell line EC1	-	Downregulation of MMP; upregulation of p21, p53, CYT-c, Bim, and cPARP levels; and activation of Caspase-3 expression, suppressing tumor growth.	Apoptosis	[84]
	Colon cancer	Human Colon carcinoma cell line HCT15	MAPK/ERK axis	The reflection of BAX and Caspase-3 was decreased, while the reflection of Bcl-2 was increased, inducing apoptosis of cancer cells.	Apoptosis Proliferation	[85]
	Pancreatic cancer	Human Pancreatic carcinoma cell line Panc-1	-	Blocked S stage stagnation, activated the cleavage of Caspase-3/9 and PARP and facilitated cell apoptosis.	Apoptosis	[86]
	Pancreatic cancer	Human Pancreatic carcinoma cell line Panc-1	MMP-2/16 axis	Downregulated MMP2 and MMP16 and inhibited cell invasion and migration.	Invasion Migration	[87]
	Liver cancer	Human Liver carcinoma cell HepG2	NF-κB axis	Downregulated Bcl-2 and upregulated BAX, Caspase-3, MMP2 and MMP9.	Migration Invasion Proliferation	[88]
	Gastric cancer	Gastric carcinoma cell line SGC-7901	-	Downregulated Bcl-2, EGFR, Akt, p-Akt and NF-κ, and upregulated BAX and Caspase-3, inducing apoptosis of cancer cells.	Apoptosis	[89]

**Table 2:** Possible mechanism of the detoxification of active ingredients in *P. vulgaris*.

Active Ingredient	Disease	Experimental Model	Mechanism	Effect	References
Oleanolic Acid	Lung injury	NMDA-induced acute lung injury in mice.	Lower NF-κB, NLRP3 and BAX upregulate the levels of SIRT1, Nrf2 and Bcl-2 proteins and reduce lung injury.	Inflammation Oxidative stress Apoptosis	[94]
	Liver injury	CCl4-induced liver injury in rats	The levels of GSH and SOD in mice with liver injury were upregulated, and the antioxidant defense system of liver was enhanced to reduce liver injury.	-	[95]
	Liver injury	CCl4-induced liver injury in rats	Downregulate MDA, ALT and AST and upregulate GSH to alleviate liver toxicity.	Inflammation	[96]
Stigmasterol	Colitis	DSS-induced colitis mouse model	Downregulated levels of TNF-α, IL-6, IL-1β, CSF-1 and COX-2; inhibited NF-κB pathway; and alleviated colitis.	Inflammation	[97]
Luteolin	Liver and kidney injury	Doxorubicin-induced hepatic and renal disorders in rats	The antioxidant capacity and IL-10 level were improved, the activity of caspase-3/9 was reduced and hepatorenal toxicity was alleviated.	Inflammation Apoptosis	[98]
	Liver and kidney injury	Methotrexate-induced hepatorenal toxicity in rats	The expressions of Nrf2, GSH, CAT and Bcl-2 were upregulated, and the expressions of ROS, NF-κB and BAX were downregulated to reduce hepatorenal toxicity.	Inflammation Apoptosis	[99]
	Liver injury	CCl4-induced liver injury in mice	TNF-α, IL-6, IL-1β, Caspase-3 and BAX were downregulated, and Bcl-2 was upregulated, reduce toxicity and protect ing against liver damage.	Inflammation Apoptosis	[100]
Rosmarinic Acid	Liver and kidney injury	Methotrexate-induced hepatorenal toxicity in rats	Upregulation of GSH and CAT, downregulation of MDA, reduction in degeneration and cell vacuolization in liver tissue and reduction in hepatorenal toxicity.	Inflammation	[101]
	Liver and kidney injury	Cisplatin-induced liver and kidney injury in mouse model	Downregulation of ALT, AST, BUN and CRE levels and inflammatory factor IL-1 β, IL-6 and TNF-α, activating the Nrf2 signaling pathway to prevent cisplatin induced liver and kidney damage.	Inflammation	[102]
	Liver and kidney injury	CCl4-induced liver injury in mice	Downregulated ALT, ALP, Caspase-3, TG, TC, MDA, TNF-α, IL-6 and IL-8 and upregulated GSH, SOD, CAT and Nrf2 levels to alleviate liver and kidney damage.	Inflammation Apoptosis	[103]
	Liver injury	Paracetamol-induced hepatotoxicity in rats	MDA, ALT and AST were decreased, while TAC, GSH and GST were increased, reducing liver damage.	Inflammation	[104]

## Abbreviations

OB	Oral Availability
DL	Drug-like properties
KEGG	Kyoto Encyclopedia of Traditional Chinese Medicine
TC	Total Cholestero
LDL-C	Low-density lipoprotein cholesterol
HDL-C	High-density lipoprotein cholesterol
BAX	Bcl-2-associated X
Bcl-2	B-cell lymphoma-2
VEGF	Vascular endothelial growth factor
NF-κB	Nuclear factor kappa-B
PI3K	Phosphatidylinositol 3-kinase
AKT	Protein kinase-B
JAK	Janus Kinase
STAT	Signal transducer and activator of transcription
ROS	Reactive oxygen species
PERK	PKR-like endoplasmic reticulum kinase
ATF4	Recombinant activating transcription factor 4
APAF-1	Apoptotic protease activating factor-1
NF-κB	Nuclear factor kappa-beta
MEK	Methyl ethyl ketone
ERK	Extracellular regulated protein kinases
JNK	C-Jun N-terminal kinase
VEGFR2	Vascular endothelial growth factor receptor 2
PCNA	Proliferating cell nuclear antigen
PARP	Poly-ADP-ribose polymerase
mTOR	Mechanistic target of rapamycin
LC3-II	Iight chain 3-II
XIAP	X-linked inhibitor of apoptosis protein
Jab1	Jun activation domain-binding protein 1
cMet	C-mesenchymal-epithelial transition
PARP-1	Poly-ADP-ribose polymerase 1
FOXO3a	Forkhead box O3a
Nrf2	Nuclear factor erythroid-2 related factor 2
HO-1	Heme oxygenase-1
MMP	Matrix metalloproteinase
CYT-c	Cytochrome C
MAPK	Mitogen-activated protein kinase
ERK	Extracellular signal-regulated kinase
MMP2	Matrix metallopeptidase 2
MMP16	Matrix metallopeptidase 16
MMP9	Matrix metallopeptidase 9
AMPK	AMP-activated protein kinase
IL-6	Interleukin 6
IL-8	Interleukin 8
IL-10	Interleukin 10
TNF-α	Tumor necrosis factorα
COX-2	Cyclo-oxygenase-2
TGF-β1	Transforming growth factor-β1
α-SMA	α-Smooth muscle actin
NLRP3	NOD-like receptor thermal protein domain associated protein 3
SIRT1	Recombinant Sirtuin 1
GSH	Glutathione
SOD	Superoxide dismutase
MDA	Malondialdehyde
ALT	Alanine transaminase
AST	Aspartate transaminase
CSF-1	Macrophage colony-stimulating factor 1
CAT	Catalase
IL-1β	Interleukin—1β
ALP	Alkaline phosphatase
TG	Triglyceride
